# IL-32: A Novel Pluripotent Inflammatory Interleukin, towards Gastric Inflammation, Gastric Cancer, and Chronic Rhino Sinusitis

**DOI:** 10.1155/2016/8413768

**Published:** 2016-04-06

**Authors:** Muhammad Babar Khawar, Muddasir Hassan Abbasi, Nadeem Sheikh

**Affiliations:** ^1^Cell and Molecular Biology Lab, Department of Zoology, University of the Punjab, Lahore 54590, Pakistan; ^2^Department of Zoology, Government College of Science, Wahdat Road, Lahore, Pakistan

## Abstract

A vast variety of nonstructural proteins have been studied for their key roles and involvement in a number of biological phenomenona. Interleukin-32 is a novel cytokine whose presence has been confirmed in most of the mammals except rodents. The IL-32 gene was identified on human chromosome 16 p13.3. The gene has eight exons and nine splice variants,* namely*, IL-32*α*, IL-32*β*, IL-32*γ*, IL-32*δ*, IL-32*ε*, IL-32*ζ*, IL-32*η*, IL-32*θ*, and IL-32s. It was found to induce the expression of various inflammatory cytokines including TNF-*α*, IL-6, and IL-1*β* as well as macrophage inflammatory protein-2 (MIP-2) and has been reported previously to be involved in the pathogenesis and progression of a number of inflammatory disorders,* namely*, inflammatory bowel disease (IBD), gastric inflammation and cancer, rheumatoid arthritis, and chronic obstructive pulmonary disease (COPD). In the current review, we have highlighted the involvement of IL-32 in gastric cancer, gastric inflammation, and chronic rhinosinusitis. We have also tried to explore various mechanisms suspected to induce the expression of this extraordinary cytokine as well as various mechanisms of action employed by IL-32 during the mediation and progression of the above said problems.

## 1. Introduction

Cytokines are low molecular weight polypeptides/proteins involved in maintenance of homeostatic function in the body. They are pleotropic and are either autocrine (itself), paracrine (nearby cells), or endocrine (distant target cells) in nature [1, 2] and can be classified on the basis of their origin and biological activity. Cytokines generally include lymphokines, monokines, chemokines, and interleukins. The aim of the current review is to emphasize the existing therapeutic potential and future perspective of interleukin-32 (IL-32). The pluripotency and remarkable roles of this interleukin have encouraged their vast application in the field of medical biology.

## 2. Review

Interleukin-32 (IL-32), a recently described novel proinflammatory cytokine primarily expressed by natural killer (NK) cells, monocytes, epithelial cell lines, and T-cells, has gained popularity because of its involvement in many crucial biological phenomena [3–6]. Since its discovery in 1992, IL-32 was named natural killer cell transcript 4 (NK4) because of its selective expression in IL-2 activated NK cells [7]. The potential biological activities of IL-32 remained veiled until 2005 when Kim and coworkers (2005) reported its involvement in inducing some other inflammatory cytokines, namely, IL-8 and TNF-*α* [4]. Because of this reason a new name, IL-32, was assigned to NK4.

One problem that still remains associated with IL-32 is the identification of cell surface receptor of IL-32. The structure of IL-32 is unique in this regard as it lacks any specific cell surface receptor involved in signal transduction exterior to the cell, and it is suspected to exert its influence through some intracellular molecular interactions [8–11]. Studies have been carried out for extracellular activities of IL-32 which have suggested the integrin signaling to be involved in mediating the effects of IL-32 [12]. Recently, IL-32, via interactions with some other molecules, has been reported to be involved in various intracellular signaling. IL-32*α* being intracellularly mediated interacts with not only paxillin, protein kinase c (PKC), and integrin, but also focal adhesion kinase 1 (FAK 1). Interaction with PKC leads to modulation of IL-6 in myelomonocytes actively expressing IL-32*α* [9, 13]. Recent studies have revealed the interactions among different isoforms of IL-32 [10, 14] as well as with other transcriptional regulators and various proteins [9, 11].

One thing that is unique to this cytokine is that a significant amount of recombinant IL-32 protein is required to activate specific cells compared to that of other cytokines. That is the reason it does not seem to be a normal cytokine and does not belong to any of the known cytokine families [4].

One thing that acts as hurdle in the development of IL-32 research and* in vivo* studies is that IL-32 gene has not been identified in rodents [15]. So, most of the studies that aimed to understand the biological activities and functioning of different isoforms have been carried out using Tg mice [16].

IL-32 gene was found to be located on human chromosome 16p13.3 and was reported to exist in nine different isoforms by mRNA alternative splicing including IL-32*α*, IL-32*β*, IL-32*γ*, IL-32*δ*, IL-32*ε*, IL-32*ζ*, IL-32*η*, IL-32*θ*, and IL-32s [4, 14, 17], tagged with specific activities and properties [10, 14, 18–20]. For example, IL-32*β* is involved in increasing the immune cell adhesion to the activated endothelial cells [21]. Similarly, IL-32*γ*, which is biologically the most active one, has shown a potent antiviral role against a number of familiar viruses, namely, vesicular stomatitis virus (VSV), HIV, influenza A virus (IAV), and herpes simplex virus 2 (HSV-2) [22–26]. IL-32 has been proved as a pluripotent cytokine. Kang et al. (2013) previously reported the upregulation of IL-10 by IL-32*β* through PKC*δ* pathway [11]. In another study, Kang et al. (2013) studied the effects of other isoforms of IL-32 in IL-10 upregulation. They reported inhibitory mechanism of IL-32*δ* and its involvement in the decrease of IL-10 production. IL-32*δ* interacts with IL-32*β* and showed its inhibitory effects by suppressing the binding of IL-32*β* to PKC*δ*. Their data was indicative of regulation of IL-32 by its own isoforms [10].

Recently, interaction of IL-32*θ* with chemokine C–C motif ligand 5 (CCL5) has also been reported. Bak et al. (2014) reported that IL-32*θ* by mediating the phosphorylation of STAT3 on Ser727 downregulates CCL5 expression by interacting with PKC*δ* and renders this chemokine transcriptionally inactive. This report suggested the role of IL-32*θ* as an intracellular modulator of inflammation [27].

Recently, Kim et al. (2014) reported the involvement of IL-32*θ* in the reduction of leukemia. This isoform compared to IL-32*β* was reported to downregulate the process of differentiation of a monocytes cell line to macrophages induced by phorbol 12-myristate 13-acetate (PMA) treatment. Expression of this cytokine not only inhibited the adhesion capability of THP-1 cells (monocytic leukemia cells) either to vascular endothelial cells or to culture plates but also inhibited morphological changes. Even after the treatment of PMA in THP-1/IL-32*θ* cells, IL-32*θ* was found to inhibit the expression of various macrophage markers, namely, CD11b, CD18, and CD36. Furthermore, in THP-1/IL-32*θ* cells, there was found a reduction in the expression of PU.1 as compared to THP-1/IL-32*β* and wild type cells. Hence, IL-32*θ* was reported to inhibit monocytic differentiation by reducing the PU.1 expression [28].

In another study on acute myeloid leukemia (AML), Kim et al. (2015) described the effects of IL-32*θ* on TNF-*α* production. About 38% of total subjects of AML were found to express endogenous IL-32*θ*, compared to healthy subjects who did not even express it. To measure serum level of various proinflammatory cytokines, namely, TNF-*α*, IL-1*β*, and IL-6, plasma was assorted into two groups with or without IL-32*θ*. IL-32*θ* expressing group showed a decrease in TNF-*α* production compared to group without IL-32*θ*. It was also reported that, in an IL-32*θ* stable expression system, IL-32*θ* attenuated the PMA-induced TNF-*α* production in leukemia cell lines [29]. Bak et al. (2016) reported an inhibition of invasion and migration of colon cancer cells both* in vitro* and* in vivo* by ectopic expression of IL-32*θ*. In HT29 colon cancer cells, IL-32*θ* attenuated the invasive and migratory potential by suppressing the epithelial-mesenchymal transition (EMT). Furthermore, this interleukin is involved in alterations of a number of properties of cancer stem cells (CSCs) by inhibiting the STAT3-ZEB1 pathway and could be a tumor suppressor [30].

Previous studies carried out on the expression of IL-32 have revealed that a variety of stimuli stimulate IL-32 genes; as a result, IL-32*α* is expressed in various inflammatory cells, namely, NK cells, T-cells, peripheral blood mononuclear cells (PBMCs), and monocytes as well as some nonimmune cells, namely, endothelial cells, fibroblasts, and keratinocytes [7, 31–33].

All isoforms of IL-32 as well as IL-1*β* do not possess a classic signal peptide. Hence, because of this reason it is secreted in the least quantities and supernatant does not contain any detectable amount of IL-32. On the contrary all the measurable IL-32 was found in cell lysates [17, 26, 31, 34]. As these secreted proteins are not easily purified and their structure has not been completely evaluated, no structural data have been reported.

Initially, NK4 cDNA was not observed expressing as recombinant protein because it was found to contain a signal peptide sequence that lacks any transmembrane domain [7]. So, the NK4 coding protein was assumed to be a secreted protein. The newly discovered isoforms do not possess a putative signal peptide; therefore, the role of this interleukin still remains controversial whether it affects the cells from inside or outside [17]. Kim et al. (2005) reported that IL-32 secreted from the cells is very small in amount and difficult to detect compared to the amounts present in cytosol [4].

**Figure 1:**
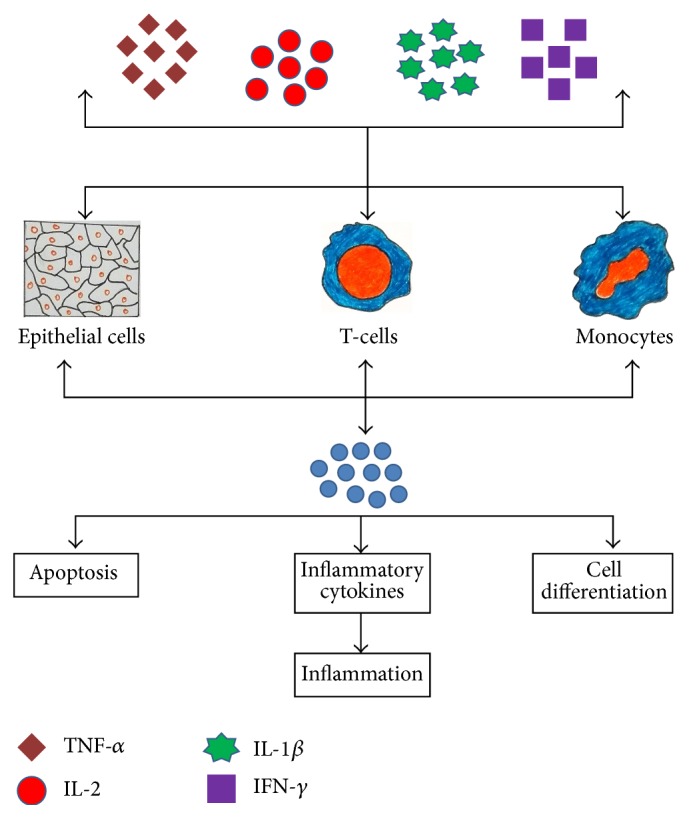
Mechanism of synthesis of IL-32 by different body cells (T-cells, epithelial cells, and blood monocytes) induced by various cytokines. IL-32 after its secretion affects a number of biological activities, namely, apoptosis and cell differentiation as well as mediation of inflammation.

IL-32 is reported to be involved in the stimulation and production of macrophage inflammatory protein-2 (MIP-2) as well as various chemokines and inflammatory cytokines, namely, IL-1*β*, IL-6, IL-8, and TNF-*α* [4, 35–37]; hence, it acts as a key player in innate and adaptive immunity* in vitro* [3]. IL-1*β*, TNF-*α*, IL-2, or IFN-*γ* is found to be involved in the production of this interleukin in epithelial cells and blood monocytes [4, 7, 38]. It is pleiotropic in action and controls cell differentiation [35, 39, 40], stimulation of pro- or anti-inflammatory cytokines [41–43], and cell death, especially apoptosis [17, 32], and is pleiotropic in the pathogenesis of various disorders, like infectious, cancerous, inflammatory, and allergic diseases (Figure 1).

Role of this cytokine in rheumatoid arthritis, chronic obstructive pulmonary disease, and inflammatory bowel disease was reviewed previously by Khawar et al. (2015) [44]. The purpose of the current review is to analyze its role related to gastric inflammation, gastric cancer, and some other related diseased conditions.

## 3. IL-32 in Gastric Inflammation and Gastric Cancer

Globally gastric cancer is one of the leading causes of cancer-related mortality and is the 4th most common type of cancer and 2nd among all mortalities [45]. In the year 2010, in Taiwan, only gastric cancer was blamed as the 6th leading cause of cancer-related deaths [46]. Prognosis of this disease is very poor due to lack of sufficient information, so the only solution left behind is surgery. So, there is an intensive need to identify the biomarkers and elaboration of the exact mechanism involved in the pathogenicity of this disease. Similarly, some novel therapeutic remedies will be worthwhile [47].

**Figure 2:**
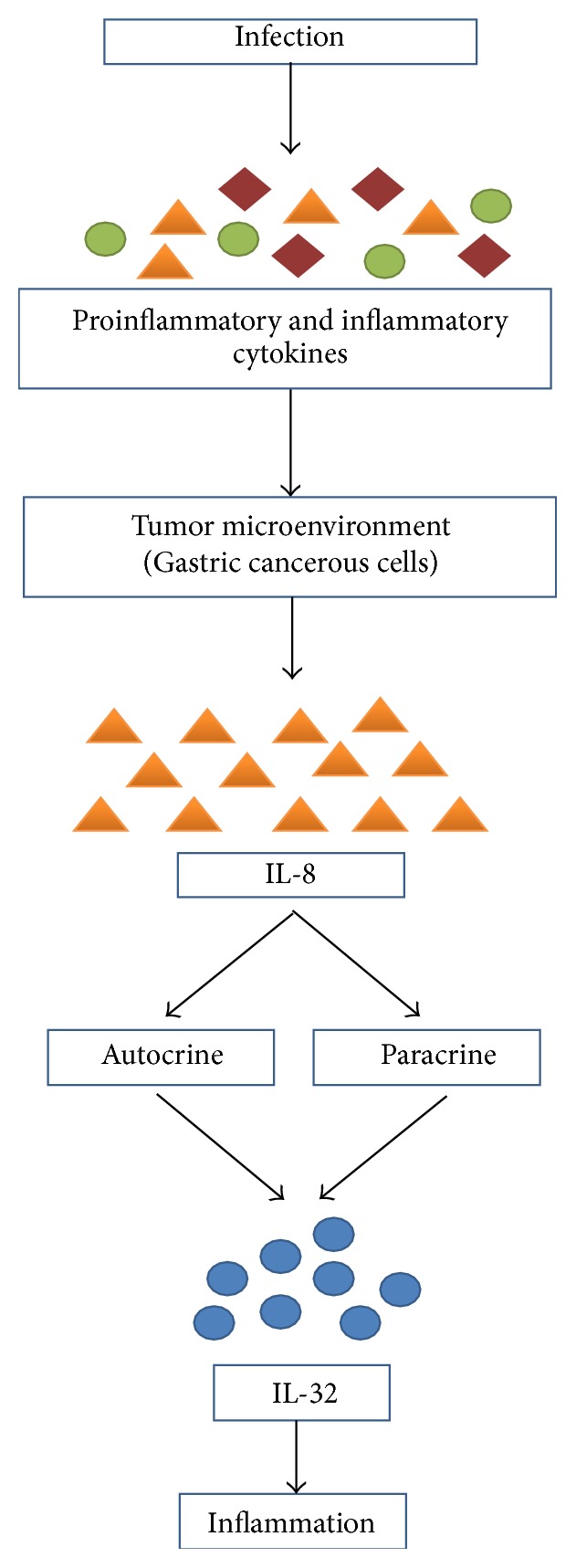
How gastric cancerous cells lead to inflammation mediated by IL-32. Infections in the body set in motion the synthesis and secretion of a variety of proinflammatory and inflammatory cytokines which results in the development of tumor microenvironment in gastric cancerous cells that are continuously involved in the production of IL-8 which exerts its biological influence by initiating the production of IL-32 which is blamed to be involved in gastric inflammation.

In response to infection, certain inflammatory cytokines are released which influence tumor production in a variety of ways. For example, gastric cancer cells secrete IL-8 which in the presence of a high level of IL-32 has been reported in mucosa of gastric cancer compared to nontumor mucosa [48] (Figure 2).

The exact mechanism involved in induction is overexpression of IL-32 which is still controversial but chromosomal region 16p13.3 was reported to be amplified and transcribed in cancer of small intestine and breast [49, 50]. Tsai et al. (2014) reported an enhanced expression of IL-32 in patients of gastric cancer which was found to be positively correlated with fierceness of the cancer. Ectopic expression of IL-32 by employing phosphor-AKT/phospho-glycogen synthase kinase 3*β*/active *β*-catenin and hypoxia-inducible factor 1*α* (HIF-1*α*) signaling pathways leads to increased invasion and cell migration as well as causing an elongated morphology by inducing the expression of MMP9, matrix metalloproteinase 2 (MMP2), VEGF, and IL-8 as well. On the contrary, by depletion of IL-32 in gastric cancer cells, all of these above-said activities were found to be reversed and lung colonization* in vivo* was found to be significantly decreased [46].

Similarly, expression of IL-32 in human stomach cancer has been studied with monoclonal antibody KU32-52 and a polyclonal antibody employing sandwich ELISA by Seo et al. (2008). The results of this ELISA were found not to react with other different cytokines, namely, hIL-2, hIL-6, hTNF-*α*, hIL-8, hIL-10, hIL-18, hIL-1*α*, and hIL-1*β*. Intra-assay coefficients and interassay coefficients variations were found as 18.5%–4.6% (*n* = 10) and 23%–9% (*n* = 10), respectively. Average serum level of IL-32 was found significantly higher in patients of stomach cancer (189 pg/mL, *n* = 16) compared to healthy control (109 pg/mL, *n* = 12) [48].

## 4. Microbial Pathogen Factors* (H. pylori)*

Inflammation and cancer related to microbial infections have been studied extensively in previous years. Robin Warren and Marshall (1983) isolated for the first time the causative agent* H. pylori* from gastritis patients [51]. Infection caused by* H. pylori* often contributes to inflammation and cancer of GIT which is one of the leading causes of death due to cancer related mortality globally [52–54]. The pathogenesis of gastric insults involves a cluster of more than 30 genes more commonly known as the* cag* pathogenicity island (PAI) of* H. pylori* which has been associated with gastric mucosa-associated lymphoid tissue (MALT) lymphoma, gastric cancer, and some other gastric diseases [55, 56].

In infected epithelial cells,* H. pylori cag*PAI activates the NF-*κ*B and mitogen-activated protein kinase (MAPK) signaling pathways. NF-*κ*B regulates various cellular responses, namely, inflammation, cell death, cell proliferation, and cell survival as well as heightening the production of other inflammatory cytokines such as interleukin-1*β* (IL-1*β*), IL-8, and tumor necrosis factor alpha (TNF-*α*). IL-8, an inflammatory cytokine, plays an important role in gastritis and gastric carcinogenesis [57–59] and induced gastric inflammation by introducing neutrophils infiltration. Increased risk of atrophic gastritis and gastric cancer in Japanese population is because of upregulation of IL-8 as a result of single polymorphism in the gene of IL-8 [60]. Polymorphisms of other inflammatory cytokines, namely, IL-1*β* and TNF-*α* gene, also have been linked with gastritis and gastric cancer [61, 62].

**Figure 3:**
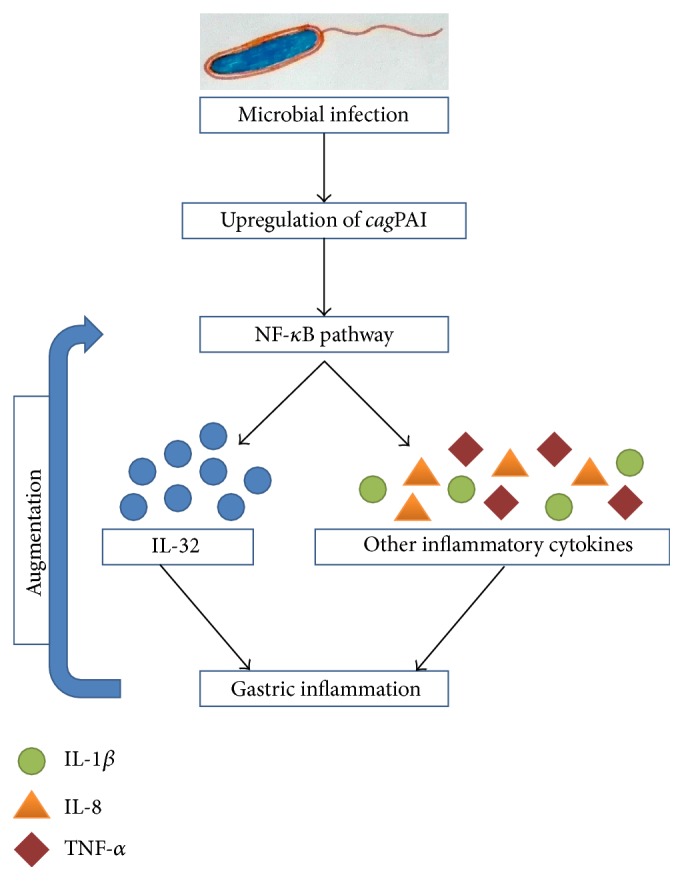
Microbial infection induced expression of IL-32 results in gastric inflammation. Microbial infection (*H. pylori*) leads to the upregulation of* cag*PAI cluster of genes which through the activation of NF-*κ*B pathway results in the synthesis of IL-32 and some other potent inflammatory cytokines (IL-1, IL-8, and TNF-*α*) that results in gastric inflammation. The resulting inflammation self-augments the activation of NF-*κ*B pathway which further aggravates the situation.

Sakitani et al. (2012) reported an elevation in the level of expression of IL-32 in human gastritis and gastric cancer tissues. Results of the following study suggested the basic role of IL-32 in gastric inflammation caused by* H. pylori* which induces NF-*κ*B activation in a* cag*PAI-dependent manner required for IL-32 upregulation in gastric tissues. Intracellular IL-32 produced in such a way augments NF-*κ*B activity and cytokine production, accelerating the inflammatory responses of gastric tissue infected with* H. pylori* [63] (Figure 3).

## 5. IL-32 in Chronic Rhinosinusitis

Chronic rhinosinusitis (CRS) referred to a diverse inflammatory disease of the nose and paranasal sinuses characterized by at least 12 weeks of two of the following symptoms: hyposmia/anosmia, nasal obstruction, mucopurulent drainage, and facial pain/pressure [64]. This problem is diagnosed on the basis of clinical history and nasal endoscopy or computed tomography scan is often used to measure inflammation. Chronic rhinosinusitis (CRS) is one of the commonest types of chronic diseases which affects about 15% of the total population globally. This problem not only stresses healthcare systems by imposing huge financial burdens but also affects the quality of life [65, 66]. The exact mechanism of pathogenesis involved in the development of CRS is still controversial and is being explored, but a number of factors are normally suspected to be involved in the development of this problem, namely, staphylococcal infection as well as cross-linking between its toxins and IgE [67, 68]. The pathogenesis of this multifaceted inflammatory disorder could be due to genetic and environmental factors [69]. Similarly, several other factors which have been previously reported specifically to be involved in the development of CRS include rhinovirus infections [70], bacterial biofilms [71], and allergic inflammation [72].

CRS with nasal polyps (CRSwNP) and CRS without nasal polyps (CRSsNP) are two common subclasses on the basis of gross endoscopic appearance. Mechanisms of inflammation and symptomatic overlap exist among these two forms while CRSwNP is distinguished as Type 2 helper T [Th2] polarized disease with marked eosinophilia in tissues than CRSsNP (more Type 1 helper T [Th1]) in whites [73].

Keswani et al. (2012) and Soyka et al. (2012) have published two different studies regarding the expression of IL-32 in primary nasal epithelial cells by inflammatory cytokines, namely, TNF-*α*, IFN-*γ*, and dsRNA (a TLR3 ligand) which stimulated the upregulation of IL-32 mRNA during CRS. IL-1*β*, IL-4, IL-13, IL-17A, IFN-*β*, peptidoglycan (a TLR2 ligand), and LPS (a TLR4 ligand) were found to have no effect on the expression of mRNA of IL-32. The presence of IL-32 protein in cell lysates of affected cells and absence in supernatants clearly showed that IL-32 is an endogenous regulator in epithelial cells. The results of both* in vitro* studies were almost similar and suggested that IL-32 was greatly reproducible as it was found upregulated in airway epithelial cells [74, 75].

Immunohistochemical analysis also confirmed the presence of IL-32 in glandular and mucosal epithelium and submucosal inflammatory cells. But no difference was found among the staining intensities of controls and epithelial cells of CRSwNP or CRSsNP [74]. Hence, there is a need for development of a more sensitive assay to detect IL-32 elevation in CRSsNP epithelial cells. Immunohistochemical analysis does not seem to be a sensitive and quantitative approach.

IL-32-positive inflammatory cells were found to be elevated in nasal polyps as well. Colocalization of IL-32 with CD68^+^ macrophages and CD3^+^ T-cells was confirmed through immunofluorescence studies which was a clear indicative of positive correlation of its expression with CD3 and macrophage mannose receptor in nasal polyps. These findings showed the involvement of macrophages and T-cells in the production of IL-32 and infiltration of the same cells is responsible for IL-32 elevation in nasal polyps [74]. Presence of IL-32 was reported in some glandular tissues of nasal polyps using immunofluorescent staining though its source was not confirmed as to whether it was coming from unfiltered inflammatory cells or from glandular epithelium [75]. Previously, IL-32 induction has been reported by Th1-related inflammatory cytokines which represents the involvement of IL-32 in Th1-mediated inflammation. However, its enhanced expression, which was characteristically more Th2-mediated type, has also been reported in sinonasal of CRSwNP type [75, 76]. Similarly, many of Th2-mediated disorders including asthma, allergic rhinitis, atopic dermatitis, and some other diseases have also been linked to IL-32 [77–79]. Severe forms of these disorders are also related to Th1 and Th2 inflammation as well [80, 81].

**Figure 4:**
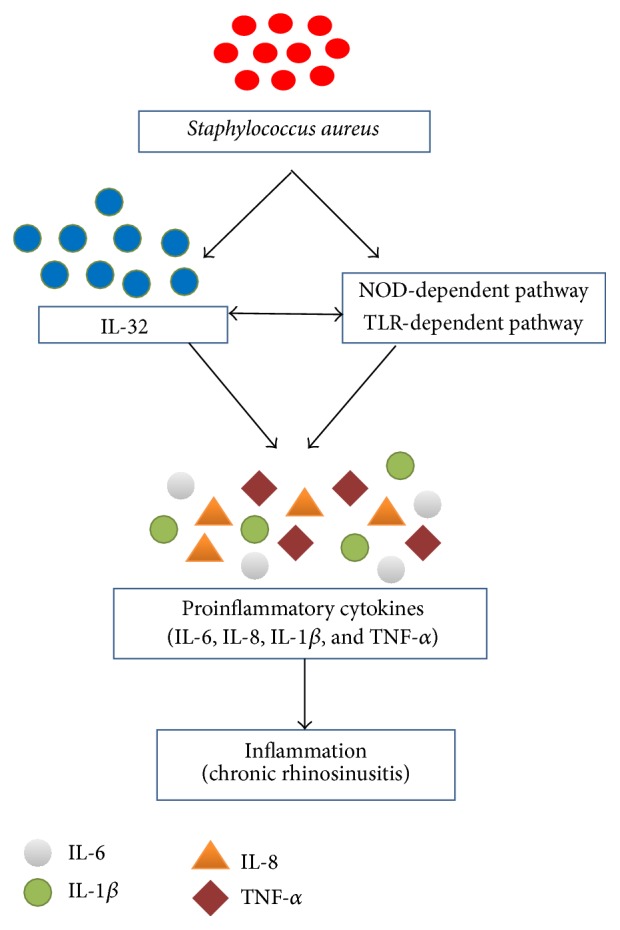
Involvement of* Staphylococcus aureus* in IL-32 induced inflammation in chronic rhinosinusitis.* Staphylococcus aureus* induces the expression of IL-32 as well as initiating the NOD-dependent pathway and TLR-dependent pathway which synergistically stimulate the production of some potent proinflammatory cytokines that results in inflammation of chronic rhinosinusitis.

IL-32 plays a prime role in the progression of CRS because of its dual property of not only acting as a proinflammatory cytokine, but also regulating a number of other important cytokines. According to one explanation, amplification of the inflammatory response pathogenesis of CRS infection is IL-32 related. Various proinflammatory cytokines, namely, IL-6 and IL-8, have been enhanced in synergism with TLR2-dependent as well as NOD-dependent pathways in nasal polyps [82–85]. Interestingly, TLR2 and NOD ligands containing* Staphylococcus aureus* commonly colonize the nasal cavity which has the capability of synergizing with IL-32 in CRSwNP type [67, 82, 86]. IL-32 is even found to be involved in the induction of a large array of inflammatory cytokines including IL-1*β*, IL-6, and IL-8, as well as TNF-*α* directly which clearly suggests the involvement of IL-32 in the pathogenesis and progression of CRS [4, 26, 37, 87] (Figure 4).

In view of the above discussion, it could be concluded that still there is a need for more intensive research to completely understand the crosstalk between IL-32 and immune system but on the basis of the available data it can be concluded that IL-32 may play a role in the progression of inflammatory response observed in case of CRS.

## 6. Conclusion

The role of cytokines towards inflammation and modulation of various infectious diseases is a hot topic today. In conclusion, IL-32 is a key modulator in the pathogenesis of various clinical problems which is mostly induced by IL-8 and enhances the severity of gastric inflammation, gastric cancer, and chronic rhino sinusitis. It not only contributes in the development of inflammation but also induces the expression of a cascade of some potent inflammatory cytokines. There is a need for further investigations of the different pathways regulated by IL-32 which will in turn allow the identification of potential targets for the prevention and treatment of autoimmune, infectious, and inflammatory diseases.
